# Evaluating the baseline hemoglobin, albumin, lymphocyte, and platelet (HALP) score in the United States adult population and comorbidities: an analysis of the NHANES

**DOI:** 10.3389/fnut.2023.1206958

**Published:** 2023-05-18

**Authors:** Ryan Antar, Christian Farag, Vincent Xu, Arthur Drouaud, Olivia Gordon, Michael J. Whalen

**Affiliations:** ^1^Department of Urology, George Washington University School of Medicine, Washington, DC, United States; ^2^Department of Medicine, George Washington University School of Medicine, Washington, DC, United States

**Keywords:** HALP score, comorbidities, immunonutrition (IN), prognostic, biomarker, cancer, nutritional status, National Health and Nutrition Examination Survey (NHANES)

## Abstract

**Introduction:**

As a composite immunonutritional biomarker, the Hemoglobin, Albumin, Lymphocyte, Platelet (HALP) score has shown promise in assessing a patient's overall health status by integrating several routinely collected laboratory indicators. This biomarker has been examined in many different populations of patients and disease states (i.e., cancer), but an integrated, universal rubric using standardized thresholds has not thus far been developed. Pre-existing large population-based databases represent an ideal source to examine the distribution of HALP and the influence of diverse health statuses on this score.

**Methods:**

We conducted a cross-sectional study using data from the National Health and Nutrition Examination Survey (NHANES) between 2017–2020, evaluating 8,245 participants across numerous demographic, socioeconomic, and health-related variables. Univariate and multivariate linear regression analyses assessed the associations between HALP scores and these factors.

**Results:**

Our findings revealed significant associations between HALP scores and various demographic, socioeconomic, and health conditions. The median HALP score among the representative population was 49.0, with varying median scores across different groups and normal reference ranges for males and females. Multivariate regression analysis showed that anemia treatment, age over 65 years, weak/failing kidneys, and cancer were independent risk factors associated with lower HALP scores. Male participants demonstrated higher HALP scores than female participants, and age was inversely related to HALP. Moreover, HALP scores were negatively associated with the number of comorbidities.

**Conclusion/discussion:**

This study set out to explore the HALP score from a population-based perspective, uncovering notable associations that offer vital insights into the score's clinical relevance and future applications. By determining a median HALP score of 49.0 and normal reference ranges within our diverse, representative sample, we establish a robust foundation for researchers to refine optimal HALP applications and thresholds. Considering the growing focus on personalized medicine, HALP holds promise as a prognostic tool, enabling clinicians to comprehend their patients' immunonutritional status better and deliver customized care.

## Introduction

Chronic inflammation and malnutrition can lead to a state of immunosuppression, heightening the risk of infection and mortality in patients suffering from chronic conditions. The Hemoglobin, Albumin, Lymphocyte, Platelet (HALP) score is an immunonutritional biomarker that integrates several routinely collected indicators to reflect a patient's overall health status in a single composite score. Developed by Chen et al. (1) to predict gastric carcinoma prognosis, the HALP score is calculated by hemoglobin (g/L) × albumin (g/L) × lymphocytes (/L)/platelets (/L), where theoretical thresholds of low scores denote poorer immunonutritional status. A clinically relevant universal threshold is yet to be determined and agreed upon.

Components of the HALP score can provide critical insight into a patient's immunonutritional status. Measuring hemoglobin levels is an important marker for anemia, a common condition exacerbated by various inflammatory processes. Albumin is the liver's negative acute-phase reactant protein, where low serum levels reflect an inflammatory state. Traditionally, albumin has also been used as a nutritional marker, although controversy exists surrounding its true utility as a nutritional biomarker. Lymphocytes and platelets are essential mediators of immune function, where low lymphocyte and high platelet levels may indicate impaired immunity and an increased risk of infection. Hence, researchers worldwide are interested in harnessing inflammatory and nutritional measurements to predict outcomes and stratify risks for patients with various conditions and perioperatively. The HALP score was developed to provide a combined assessment of these factors, showing promise in its prognostic utility in numerous cancers (2). However, the lack of a standardized threshold for this score has led to considerable heterogeneity in disease-specific HALP thresholds among different study cohorts. Moreover, HALP has yet to be examined in a population-based study representative of the diversity of health statuses in the United States.

To our knowledge, this study is the first to explore the baseline HALP status within a large, representative sample of the United States (U.S.) adult population, hoping to establish standardized thresholds for future research. Examining HALP at the population level allows this study to evaluate the HALP score in a real-world and generalizable context, yielding invaluable insights into its associations with numerous comorbidities and potential implications for disease management. Furthermore, if HALP is ever to be used for clinical decision-making, it is essential to put the score in the context of a patient's individual comorbidities. By delineating these connections and the current distribution of the HALP score in a large national dataset, this study holds the potential to define meaningful thresholds to inform clinical decision-making.

## Materials and methods

The National Health and Nutrition Examination Survey (NHANES) provides comprehensive information about the health and nutritional status of the U.S. population through a multitude of cross-sectional interviews, surveys, and physical examinations. This representative survey consists of a complex, multistage, probability sampling methodology to formulate a wealth of information about the general U.S. population's health status. In this study, we evaluated cross-sectional data collected from 2017–2020. We analyzed numerous variables, including age, sex, race, education level, income, insurance status, body mass index (BMI), c-Reactive Protein (CRP), cigarette smoking status, alcohol use, self-reported general health status, and numerous comorbidities and conditions.

The NHANES is a publicly available database, and the data collection methods of NHANES have been published elsewhere (3). Briefly following the participants' consent, NHANES-trained professional interviewers conducted in-person interviews and provided questionnaires that compiled information about the participant's demographic, socioeconomic, and health-related information. After the household interviews, participants visited the Mobile Examination Center (MEC), where various laboratory tests were performed by medical staff. Participants' nutritional status and other biochemical markers were measured by blood sampling, which was processed at the MEC to be analyzed by various collaborating laboratories nationwide. The methods used to analyze collected laboratory measurements are described in the *Laboratory Methods* section. All procedures involving research study participants were approved by the NHANES Ethics Review Board (ERB) (Protocol #2011-17 and Protocol #2018-01).

### Patient selection criteria

We excluded participants who were under the age of 18, had HALP scores >1,000, and were missing hemoglobin, albumin, lymphocyte, or platelet measurements that consist of the HALP score. Out of a total of 15,560 potential participants, the final sample consisted of 8,245 participants.

Participants were considered to have a comorbidity if they responded “*yes*” to at least one of the following from the NHANES Medical Conditions Questionnaire, “*Have you ever been told you had*…?” with subsequent conditions listed as *asthma, arthritis, congestive heart failure (CHF), coronary artery disease (CAD), a myocardial infarction (MI), a stroke, a thyroid problem, COPD/Emphysema, a liver condition, cancer or malignancy, weak/failing kidneys, diabetes, high blood pressure, and high blood cholesterol*.

Smoking status was assessed through the participant's response to the question, “Do you now smoke cigarettes?” Additionally, participant alcohol use was determined on a continuous numeric scale by their response to the question, “During the past 12 months, on those days that you drank alcoholic beverages, on average, how many drinks did you have? By a drink, I mean a 12 oz. beer, a 5 oz. a glass of wine, or one and a half ounces of liquor.” A complete list of participant questionnaires can be accessed using the NHANES public database.

### Laboratory methods

The Beckman Coulter DxH-800 Analyzer assessed the complete blood count, which included hemoglobin, lymphocyte, and platelet values of interest. The Roche Cobas 6000 analyzer was used to evaluate serum albumin, high-sensitivity C-Reactive Protein (hsCRP or CRP), and cholesterol. BMI was calculated by the Integrated Survey Information System (ISIS) using the participant's recorded weight and height during their MEC visit.

### Statistical analysis

Continuous variables were tested for normality through the Kolmogorov–Smirnov and Shapiro-Wilk tests with a visual inspection of P–P plots. HALP did not follow a normal distribution and is expressed as median, including the interquartile range (IQR) (25th and 75th percentiles). Differences between variable groups were assessed through Median and Mann-Whitney U non-parametric tests. Spearman's correlation was performed for correlations between continuous variables and HALP scores. Correlation analysis of non-parametric values was performed using the Spearman correlation coefficient. The diagnostic accuracy of the HALP score was assessed through receiver operating characteristics (ROC) curve formation and calculation of the area under the curve (AUC). The ideal cutoff values for Dialysis were chosen according to the Youden index, with subsequent assessment of its sensitivity and specificity. Univariable and Multivariable linear regression was performed for the outcome of a low HALP score. All statistical calculations were performed in SPSS software (version 29.0; SPSS Inc., Chicago, IL, USA). All reported *p*-values were based on two-sided hypotheses, with a *p*-value of < 0.05 considered statistically significant.

## Results

**Figure 1:**
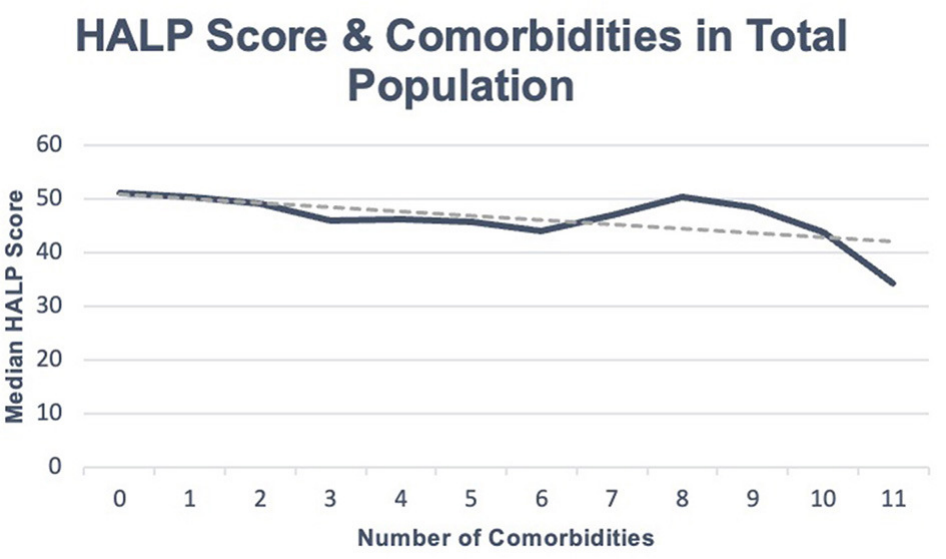
HALP score with number of comorbidities in total population.

**Figure 2:**
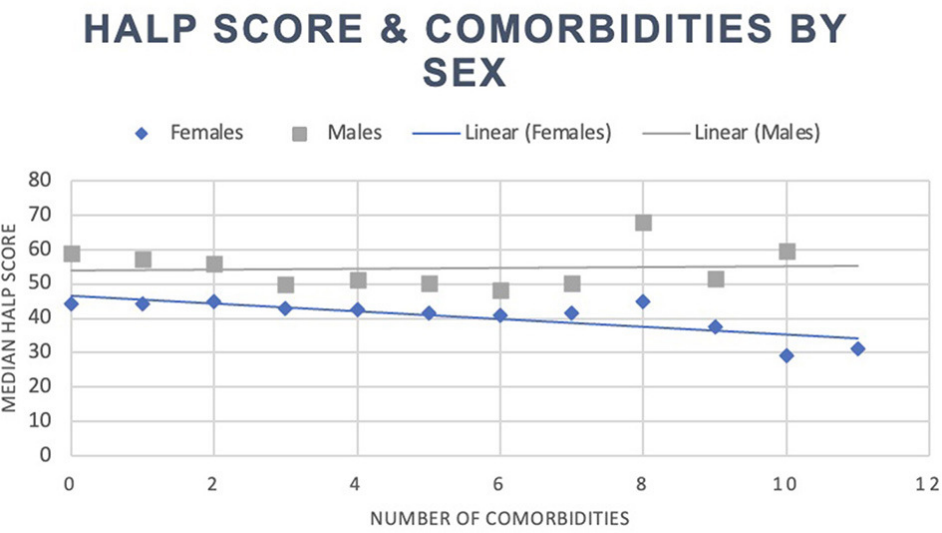
HALP score with number of comorbidities by sex.

Our total population was 8,245 participants, with a median HALP score of 49.0 (37.1–64.7). Within this sample, participants with at least one reported comorbidity had a median HALP score of 48.1 (36.2–64.3), while participants without any comorbidities had a median HALP score of 51.0 (39.4–65.6). Among the 5,758 individuals with at least one comorbidity, 2,770 (48.11%) were male and had a median HALP score of 54.1 (40.8–71.6) compared to 2,988 (51.89%) females who had a lower median HALP score of 43.6 (33.6–56.7). Similarly, 2,487 participants had no comorbidities. Of these participants, 1,232 (49.54%) were male and had a median HALP score of 59.1 (47.1–73.6) compared to the 1,255 (50.46%) female participants who had a median HALP score of 44.1 (34.2–55.8). Participants' HALP scores were further stratified by sex and number of comorbidities, as summarized in Table 1. An inverse relationship was observed between the median HALP score and the number of comorbidities in our total population (Figure 1) and in males and females (Figure 2).

**Table 1:** Total comorbidities and stratified comorbidities by sex.

**Variable**	**Median HALP score (IQR)**	**Median test**	**Mann-Whitney test**
**Total population with at least one comorbidity:**			
Yes (*n =* 5,758)	48.1 (36.3–64.0)	*p < * 0.001	*p < * 0.001
No (*n =* 2,487)	51.1 (39.3–65.6)		
**Total population with three or more comorbidities:**			
Yes (*n =* 2,580)	46.1 (34.3–61.9)	*p < * 0.001	*p < * 0.001
No (*n =* 5,665)	50.4 (38.6–65.5)		
**Total population with five or more comorbidities:**			
Yes (*n =* 831)	46.1 (33.0–60.8)	*p < * 0.001	*p < * 0.001
No (*n =* 7,414)	49.4 (37.6–65.0)		
**Males with at least one comorbidity:**			
Yes (*n =* 2,770)	54.0 (40.7–71.5)	*p < * 0.001	*p < * 0.001
No (*n =* 1,232)	59.0 (47.1–73.6)		
**Males with three or more comorbidities:**			
Yes (*n =* 1,215)	50.3 (37.1–69.0)	*p < * 0.001	*p < * 0.001
No (*n =* 2,787)	57.9 (45.1–73.0)		
**Males with five or more comorbidities:**			
Yes (*n =* 406)	50.4 (35.1–68.6)	*p < * 0.001	*p < * 0.001
No (*n =* 3,596)	56.1 (43.3–72.4)		
**Females with at least one comorbidity:**			
Yes (*n =* 2,988)	43.6 (33.6–56.7)	*p =* 0.414	*p =* 0.537
No (*n =* 1,255)	44.1 (34.2–55.8)		
**Females with three or more comorbidities:**			
Yes (*n =* 1,365)	42.4 (32.4–56.4)	*p =* 0.004	*p =* 0.140
No (*n =* 2,878)	44.3 (34.3–56.5)		
**Females with five or more comorbidities:**			
Yes (*n =* 425)	41.2 (31.2–55.0)	*p =* 0.830	*p =* 0.020
No (*n =* 3,818)	44.0 (34.1–56.8)		

Furthermore, statistically significant differences in HALP scores were found for race, age, and insurance status. Participants' education level showed borderline statistical significance, while no significant differences were found in income levels. Our study's sample included 2,073 (25.14%) black participants who had a lower median HALP score (45.8; 34.3–60.5) compared to 6,172 (74.56%) non-black participants (50.1; 38.3–65.5) (*p* < 0.001). With regards to age, 1,992 (24.16%) participants were above 65 years old and had a lower median HALP score (45.6; 34.2–60.0) than 6,253 (75.84%) participants who were under 65 years old (50.2; 38.2–65.6) (*p* < 0.001). We found that 4,496 (57.22%) participants with a college education had a lower median HALP score (48.4; 37.1–63.6) than the 3,362 (42.78%) participants with a high school diploma or less (49.6; 37.1–65.8) (*p* < 0.001). Furthermore, the median HALP score was lower in 6,910 (84.01%) insured participants (48.6; 36.6–64.2) compared to 1,315 (15.99%) uninsured participants (51.2; 39.3–66.4) (*p* < 0.001). The demographic characteristics of our sample are summarized in Table 2.

**Table 2:** Demographic characteristics.

**Variable**	**Median HALP score (IQR)**	**Median test**	**Mann-Whitney test**
**Sex**			
Male (*n =* 4,002)	55.8 (42.6–72.1)	*p < * 0.001	*p < * 0.001
Female (*n =* 4,243)	43.8 (33.8–56.5)		
**Race**			
Black (*n =* 2,073)	45.8 (34.3–60.5)	*p < * 0.001	*p < * 0.001
Non-Black (*n =* 6,172)	50.1 (38.3–65.6)		
**Age**			
< 65 (*n =* 6,253)	50.2 (38.2–65.6)	*p < * 0.001	*p < * 0.001
65+ (*n =* 1,992)	45.6 (34.2–60.0)		
**Education level**			
HS degree or less (*n =* 3,362)	49.6 (37.1–65.8)	*p =* 0.038	*p =* 0.051
Some college plus (*n =* 4,496)	48.4 (37.1–63.6)		
**Income**			
Below poverty line (*n =* 6,939)	49.0 (36.8–64.7)	*p =* 0.648	*p =* 0.281
Above poverty line (*n =* 1,306)	49.5 (38.5–64.0)		
**Insurance status**			
Uninsured (*n =* 1,315)	51.2 (39.3–66.4)	*p < * 0.001	*p < * 0.001
Insured (*n =* 6,910)	48.6 (36.6–64.2)		

Participants who had received treatment for anemia had a median HALP score of 35.4 (25.6–46.6) compared to a HALP score of 50.0 (37.9–65.3) for those who did not receive anemia treatment (*p* < 0.001). In participants with asthma, a lower median HALP score (47.0, 35.0–62.5) was observed compared to those without asthma (49.5, 37.7–65.0) (*p* < 0.001). A lower median HALP score of 46.1 (34.5–60.6) was seen in participants with arthritis compared to those without arthritis, 50.3 (38.3–65.6) (*p* < 0.001). More specifically, there was no statistical significance between participants with inflammatory arthritis (i.e., rheumatoid and psoriasis) and non-inflammatory arthritis (i.e., osteoarthritis). Participants with CHF had a median HALP score of 44.7 (31.1–59.3) compared to 49.2 (37.3–64.7) in participants without CHF (*p* < 0.001). Moreover, a lower median HALP score of 44.9 (33.8–60.4) was seen in stroke participants in contrast to 49.1 (37.3–64.7) in participants without a reported stroke (*p* < 0.001). When observing participants with reported hypertension, a median HALP score of 47.4 (35.0–63.6) was seen compared to non-hypertensive participants (49.9, 38.2–65.1) (*p* < 0.001). No statistical significance was observed between median HALP scores in CAD, angina, high blood cholesterol, and MI groups.

In participants with a reported thyroid problem, a median HALP score of 44.5 (33.7–58.7) was observed, which was lower than those without a thyroid problem (49.6, 37.6–65.2) (*p* < 0.001). Participants with COPD/emphysema had a median HALP score of 46.6 (34.1–62.7) compared to 49.1 (37.3–64.7) (*p* < 0.001). A median HALP score of 52.5 (39.8–70.2) was seen in participants with a reported liver problem in contrast to those without a liver problem (48.7, 36.8–64.1) (*p* < 0.001).

**Figure 3:**
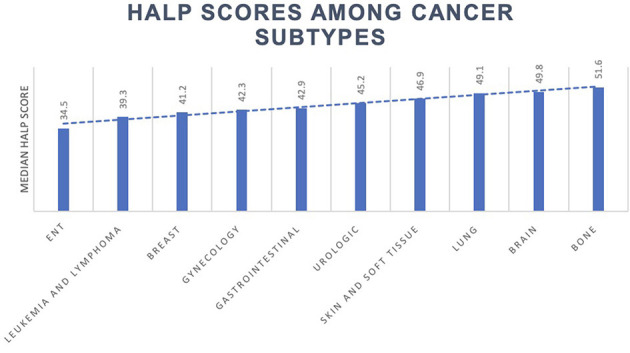
HALP score among cancer subtypes.

The median HALP score decreased in participants with cancer (43.5; 31.8–59.4) compared to those without (49.6; 37.8–65.0). Of the 819 cancer participants in this study, the most prevalent malignancy types were skin and soft tissue, urologic, breast, gynecologic, and gastrointestinal origin. Other cancer types included leukemia, lymphoma, ear, nose, and throat (ENT), bone, brain, and lung cancers. Median HALP scores among various cancer types and other conditions are summarized in Table 3 and Figure 3.

**Table 3:** Comorbidities and other factors.

**Variable**	**Median HALP score (IQR)**	**Median test**	**Mann-Whitney test**
**Have you had anemia treatment in past 3 months?**			
Yes (*n =* 430)	35.4 (25.6–46.6)	*p < * 0.001	*p < * 0.001
No (*n =* 7,797)	50.0 (37.9–65.3)		
**Have you ever been told you had asthma?**			
Yes (*n =* 1,318)	47.0 (35.0–62.5)	*p =* 0.002	*p < * 0.001
No (*n =* 6,920)	49.5 (37.7–65.0)		
**Have you ever been told you had arthritis?**			
Yes (*n =* 2,420)	46.1 (34.5–60.6)	*p < * 0.001	*p < * 0.001
No (*n =* 5,428)	50.3 (38.3–65.6)		
**Which type of arthritis was it?**			
Inflammatory (Rheum/Psoriasis) (*n =* 536)	46.9 (34.0–61.5)	*p =* 0.354	*p =* 0.157
Non-Inflammatory (Osteo) (*n =* 1,013)	45.3 (34.2–58.9)		
**Have you ever been told you had CHF?**			
Yes (*n =* 292)	44.7 (31.1–59.3)	*p < * 0.001	*p < * 0.001
No (*n =* 7,557)	49.2 (37.3–64.7)		
**Have you ever been told you had CAD?**			
Yes (*n =* 353)	50.4 (37.5–66.9)	*p =* 0.190	*p =* 0.320
No (*n =* 7,492)	48.8 (37.1–64.5)		
**Have you ever been told you had angina?**			
Yes (*n =* 206)	48.9 (38.4–65.9)	*p =* 0.944	*p =* 0.595
No (*n =* 7,628)	48.9 (37.0–64.5)		
**Have you ever been told you had a myocardial infarction?**			
Yes (*n =* 366)	48.1 (36.0–65.4)	*p =* 0.261	*p =* 0.352
No (*n =* 7,492)	49.0 (37.1–64.5)		
**Have you ever been told you had a stroke?**			
Yes (*n =* 400)	44.9 (33.8–60.4)	*p =* 0.002	*p < * 0.001
No (*n =* 7,457)	49.1 (37.3–64.7)		
**Have you ever been told you had a thyroid problem?**			
Yes (*n =* 946)	44.5 (33.7–58.7)	*p < * 0.001	*p < * 0.001
No (*n =* 6,906)	49.6 (37.6–65.2)		
**Have you ever been told you had COPD/Emphysema?**			
Yes (*n =* 719)	46.6 (34.1–62.7)	*p =* 0.015	*p < * 0.001
No (*n =* 7,135)	49.1 (37.3–64.7)		
**Have you ever been told you had a liver condition?**			
Yes (*n =* 410)	52.5 (39.8–70.2)	*p =* 0.003	*p < * 0.001
No (*n =* 7,442)	48.7 (36.8–64.1)		
**Have you ever been told you had cancer or malignancy?**			
Yes (*n =* 818)	43.5 (31.8–59.4)	*p < * 0.001	*p < * 0.001
No (*n =* 7,048)	49.6 (37.8–65.0)		
**Cancer type**			
Urologic (*n =* 169)	45.2 (33.1–63.7)		
Leukemia and Lymphoma (*n =* 29)	39.3 (27.2–59.7)		
Breast (*n =* 115)	41.2 (31.7–57.9)		
Gynecology (*n =* 87)	42.3 (31.4–53.7)		
Gastrointestinal (*n =* 61)	42.9 (28.3–52.2)		
ENT (*n =* 36)	34.5 (25.5–56.1)		
Skin and Soft Tissue (*n =* 218)	46.9 (34.6–63.7)		
Bone (*n =* 4)	51.6 (27.6–71.1)		
Brain (*n =* 2)	49.8 (49.8–49.1)		
Lung (*n =* 22)	49.1 (34.1–61.0)		
**Ever been told you have weak/failing kidneys?**			
Yes (*n =* 326)	41.6 (30.5–57.1)	*p < * 0.001	*p < * 0.001
No (*n =* 7,530)	49.2 (37.5–64.7)		
**Have you received dialysis in the past 12 months?**			
Yes (*n =* 30)	33.5 (26.3–44.3)	*p =* 0.035	*p =* 0.012
No (*n =* 296)	43.3 (31.2–58.7)		
**Have you ever been told you have diabetes?**			
Yes (*n =* 1,222)	48.5 (35.6–65.5)	*p =* 0.403	*p =* 0.168
No (*n =* 7,018)	49.1 (37.4–64.5)		
**Have you ever been told you have high blood pressure?**			
Yes (*n =* 3,045)	47.4 (35.0–63.6)	*p < * 0.001	*p < * 0.001
No (*n* = 5,188)	49.9 (38.2–65.1)		
**Have you ever been told you have high blood cholesterol?**			
Yes (*n =* 2,837)	48.6 (36.8–64.7)	*p =* 0.178	*p =* 0.569
No (*n =* 5,355)	49.4 (37.3–64.7)		
**Do you now smoke cigarettes?**			
Yes (*n =* 1,434)	55.0 (40.9–71.5)	*p < * 0.001	*p < * 0.001
No (*n =* 1,892)	49.1 (36.9–65.4)		

Participants with weak/failing kidneys were observed to have a significantly lower median HALP score of 41.6 (30.5–57.1) compared to 49.2 (37.5–64.7) in participants that did not report weak/failing kidneys (*p* < 0.001). Furthermore, participants who had received dialysis within the past 12 months of data collection had a median HALP score of 33.5 (26.3–44.3) compared to 43.3 (31.2–58.7) who had not received dialysis treatment (*p* = 0.012). There was no statistical significance between median HALP scores in diabetic and non-diabetic participants. Participants that reported smoking cigarettes at the time of data collection had a high median HALP score (55.0, 40.9–71.5) compared to non-cigarette smokers (49.1, 36.9–65.4) (*p* < 0.001).

Lastly, we evaluated the median HALP score of participants with responses to the question “Would you say your health in general is…” with responses ranging from “Excellent,” “Very good,” “Good,” “Fair,” and “Poor.” These results are shown in Table 4, demonstrating that the median HALP score decreased with more negative responses to the overall health rating. Specifically, participants who answered “Excellent” had a median HALP score of 50.6 (38.6–64.3), while those who answered “Poor” had a median HALP score of 43.9 (31.2–59.8).

**Table 4:** Rate your health: “Would you say your health in general is…”.

**Health rating**	**Median HALP score (IQR)**
1. Excellent (*n =* 934)	50.5 (38.6–64.3)
2. Very good (*n =* 2,254)	50.0 (38.0–65.0)
3. Good (*n =* 3,050)	49.1 (37.1–64.9)
4. Fair (*n =* 1,683)	48.2 (36.0–64.5)
5. Poor (*n =* 315)	43.9 (31.2–59.8)

### Univariate linear regression analysis

After conducting a series of univariate linear regression analyses, several factors were significantly associated with HALP scores. Male participants demonstrated an average 12.3-point higher HALP score than female participants, accounting for 6.5% of the variation (*p* < 0.001). On a continuous scale, age was inversely related to HALP, with a one-year increase in age associated with a 0.86 decrease in the HALP score (*p* < 0.001, R^2^ = 0.4%). Participants over the age of 65 exhibited a 4.2 decrease compared to younger individuals (*p* < 0.001, R^2^ = 0.6%). Black participants had a 4.1-point lower HALP score than non-black participants (*p* < 0.001, R^2^ = 0.6%). Additionally, participants with higher education levels (β = −1.4, *p* = 0.011, R^2^ = 0.1%) and insurance (β = −3.1, *p* < 0.001, R^2^ = 0.2%) displayed lower HALP scores. When assessing BMI on a continuous scale, a one-unit increase in BMI was associated with a 0.1 decrease in the HALP score (*p* = 0.010, R^2^ = 0.1%).

Participants' HALP scores were negatively associated with the number of comorbidities on a continuous scale (β = −0.855, *p* < 0.001, R^2^ = 0.5%). Health conditions such as anemia treatment (β = −15.8, *p* < 0.001, R^2^ = 2.1%), asthma (β = −2.4, *p* = 0.001, R^2^ = 0.1%), arthritis (β = −3.3, *p* < 0.001, R^2^ = 0.4%), congestive heart failure (β = −5.6, *p* < 0.001, R^2^ = 0.2%), stroke (β = −3.7, *p* = 0.003, R^2^ = 0.1%), thyroid problems (β = −5.4, *p* < 0.001, R^2^ = 0.5%), COPD/emphysema (β = −2.0, *p* = 0.029, R^2^ = 0.1%), cancer or malignancy (β = −5.4, *p* < 0.001, R^2^ = 0.5%), weak/failing kidneys (β = −7.2, *p* < 0.001, R^2^ = 0.4%), and hypertension (β = −1.888, *p* < 0.001, R^2^ = 0.1%) were all negatively associated with HALP scores. Participants who reported receiving dialysis in the past 12 months had an 8.3-point lower HALP score than those who did not (*p* = 0.049, R^2^ = 1.2%). Participants with a liver condition (β = 4.2, *p* < 0.001, R^2^ = 0.2%) and smoke cigarettes (β = 6.109, *p* < 0.001, R^2^ = 1.4%) were associated with higher HALP scores. Moreover, for every unit increase in CRP, the HALP score decreased by 0.328 (*p* < 0.001, R^2^ = 1.4%).

However, some factors, such as the number of alcoholic drinks per day and high blood cholesterol, did not show a statistically significant relationship with HALP scores. The results (β coefficients, *p*-values, and R^2^ values) from our univariate analysis are presented in Table 5. Select variables associated with HALP in the univariate analysis were included in further multivariate analysis.

**Table 5:** Univariate linear regression for the HALP score.

**Variable**	**Beta coefficient**	***p*-value**	**R^2^**
Sex (Male)	12.3	< 0.001	6.5%
Race (Black)	−4.1	< 0.001	0.6%
Age (continuous)	−0.86	< 0.001	0.4%
Age (Above 65)	−4.2	< 0.001	0.6%
Education Level	−1.4	0.011	0.1%
Insurance Status	−3.1	< 0.001	0.2%
BMI (continuous)	−0.1	0.010	0.1%
Have you had Anemia Treatment in past 3 months?	−15.8	< 0.001	2.1%
Have you ever been told you had asthma?	−2.4	< 0.001	0.1%
Have you ever been told you had arthritis?	−3.3	< 0.001	0.4%
Have you ever been told you had CHF?	−5.6	< 0.001	0.2%
Have you ever been told you had a stroke?	−3.7	0.003	0.1%
Have you ever been told you had a thyroid problem?	−5.4	< 0.001	0.5%
Have you ever been told you had COPD/Emphysema?	−2.0	0.029	0.1%
Have you ever been told you had a liver condition?	4.2	< 0.001	0.2%
Have you ever been told you had cancer or malignancy?	−5.4	< 0.001	0.5%
Ever been told you have weak/failing kidneys?	−7.2	< 0.001	0.4%
Have you received dialysis in the past 12 months?	−8.3	0.049	1.2%
Comorbidities (continuous)	−0.855	< 0.001	0.5%
CRP (continuous)	−0.328	< 0.001	1.4%
Alcohol drinks per day (continuous)	−0.005	0.572	0.8%
Cigarette-smoking (yes/no)	6.109	< 0.001	1.4%
High blood pressure	−1.888	< 0.001	0.1%
High blood cholesterol	0.367	0.513	0%

### Multivariate regression analysis

A multivariate regression was performed to show the predictive value of the HALP score for significant comorbidities, with several factors found to be independently associated with HALP scores. After accounting for sex, age, CRP, and smoking status, a lower HALP score was associated with an increased risk for kidney failure and cancer.

Accounting for the other variables in the model, several variables had significantly different HALP scores: (1) Anemia treatment within the last 3 months: 12.314 point difference in the HALP score (β 95% CI: −16.2, −8.3) (*p* < 0.001); (2) age >65 years old: 5.203 (β 95% CI: −7.2, −3.2) point lower HALP score.; (3) Having weak/failing kidneys: 4.934 decrease in the HALP score (β 95% CI: −8.7, −1.1) (*p* = 0.012); (4) Cancer or malignancy: 3.173 point lower HALP score (β 95% CI: −5.8, −0.53) (*p* = 0.018); (5) Increased CRP levels: a single unit increase in CRP decreases the HALP score by 0.241 (β 95% CI: −0.321, −0.160) (*p* < 0.001); (6) Cigarette smoking: 4.572 point higher HALP score (β 95% CI: 2.8, 6.3) (*p* < 0.001). Also, males were associated with a 10.829 (β 95% CI: 9.1, 12.5) point higher HALP score than females. Results from the multivariate regression analysis are presented in Table 6.

**Table 6:** Multivariate regression analysis for the HALP score.

**Variable**	**Unstandardized B**	**Significance (*p*-value)**	**95% CI for B (lower bound, upper bound)**
Anemia Treatment within the last 3 months	−12.314	< 0.001	−16.2, −8.3
Age >65 years old	−5.203	< 0.001	−7.2, −3.2
Having weak/failing kidneys	−4.934	0.012	−8.7, −1.1
Cancer or malignancy	−3.173	0.018	−5.8, −0.53
Increased CRP Levels	−0.241	< 0.001	−0.321, −0.160
Cigarette-smoking	4.572	< 0.001	2.8, 6.3
Males	10.829	< 0.001	9.1, 12.5

### Receiver operating characteristic analysis

**Figure 4:**
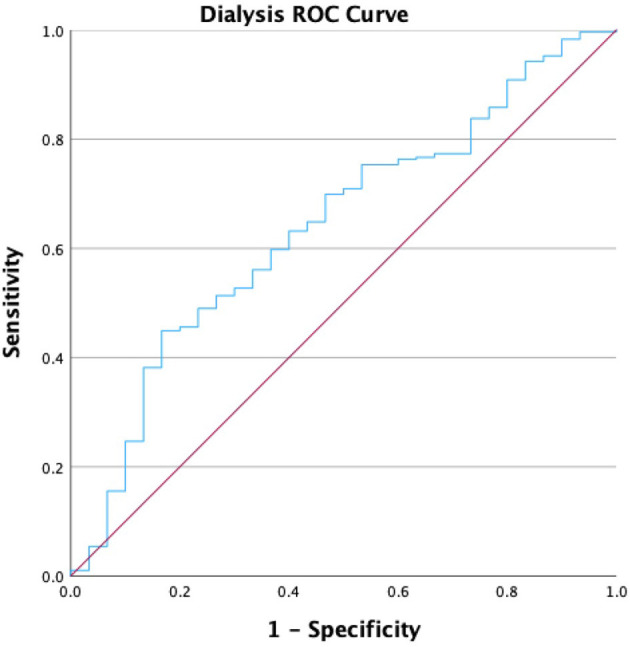
Dialysis in the past 12 months ROC curve.

We performed a receiver operating characteristic (ROC) analysis to evaluate the diagnostic ability of the HALP score for various comorbidities, and the ROC values are presented in Table 7. The area under the curve (AUC) values were highest for anemia treatment (0.721; *p* < 0.001), dialysis (0.640; *p* < 0.001), and kidney failure (0.602; *p* < 0.001). The HALP score achieved a Youden Index of 36.25, with a sensitivity of 63.2% and specificity of 60% for participants receiving dialysis in the past 12 months, as shown in Figure 4. The AUCs for CHF and stroke were 0.576 and 0.556, respectively (*p* < 0.001), while the AUC for high blood pressure was 0.534 (*p* < 0.001). Notably, there was no significant AUC for participants with high cholesterol. The AUC for participants reporting thyroid problems was 0.574 (*p* < 0.001), similar to CHF. The AUCs for respiratory conditions such as asthma and COPD/emphysema were 0.534 and 0.539, respectively (*p* < 0.001) (See Supplementary material for remaining ROC curve figures).

**Table 7:** ROC summary for the HALP score.

**Variable**	**AUC**	**Significance**	**95% CI**
Arthritis	0.558	< 0.001	0.544–0.572
Asthma	0.534	< 0.001	0.517–0.552
Kidney failure	0.602	< 0.001	0.569–0.635
Dialysis	0.640	0.012	0.538–0.741
Cancer	0.581	< 0.001	0.559–0.602
Anemia treatment	0.721	< 0.001	0.696–0.746
CHF	0.576	< 0.001	0.541–0.611
Stroke	0.556	< 0.001	0.525–0.586
Thyroid	0.574	< 0.001	0.554–0.593
COPD/emphysema	0.539	< 0.001	0.516–0.562
High blood pressure	0.534	< 0.001	0.520–0.547
High blood cholesterol	0.504	0.569	0.491–0.517

## Discussion

We investigated the HALP score in a population-based sample, observing associations between the HALP score and a diverse array of demographic factors and comorbidities. Our findings showed a significantly greater HALP score in males than females, as supported by our multivariate analysis. Given differences in established reference laboratory values in males and females, sex may influence the HALP score. Hemoglobin measures have been documented to be lower in females than males (4). Therefore, this fact lends further credence to our observation that males have a higher baseline HALP score than females. We then aimed to establish a normal reference range for the HALP score, stratified by sex. Using standardized laboratory methodology, we determined the 95% confidence intervals and calculated the reference range based on two standard deviations from the mean HALP scores for participants without comorbidities. We discovered that the mean HALP score for males was 62.1 (SD: 22), with a 95% CI of 60.9–63.3 and a reference range of 18.1–106.1. In contrast, females had a mean HALP score of 46.7 (SD: 18.1), with a 95% CI of 45.7–47.7 and a reference range of 10.6–82.8. This study is the first to establish normal HALP ranges, potentially providing a foundation for standardized baseline HALP values in patients. However, further validation with confirmatory studies can refine these values. Additionally, we acknowledge that the HALP score has proven to be highly predictive for various health conditions, suggesting that determining a normal HALP range using the lower and upper limits of two standard deviations from the mean may not be the optimal approach for this score given the large range we've described.

Furthermore, our study revealed an inverse relationship between the HALP score and age, exhibiting a decrease of 0.86 for each yearly increment in age. This association reflects the inherent decline in the critical components of HALP–hemoglobin and albumin levels—as individuals age (5). This connection also considers other potential factors, such as different age-related physiological changes, heightened vulnerability to chronic diseases, and a compromised immune system, collectively contributing to a lower HALP score. In addition, for every one-unit increase in BMI and CRP in our total population, the HALP score decreased by 0.1 and 0.241 (0.328 in univariate), respectively. Understanding the association between the HALP score and these health indices can pave the way for more effective integration of biomarkers, such as HALP, in assessing future patients.

Regarding race, our findings revealed a notable difference in median HALP scores, with black participants demonstrating a lower score (45.8; 34.3–60.5) than non-black participants (50.1; 38.3–65.5). The univariate analysis further showed black participants having a 4.1-point lower HALP score relative to non-black participants. This finding likely highlights the intricate interplay of socio-economic and social determinants of health in shaping these disparities.

Interestingly, we discovered that participants with a college education had a lower median HALP score than those with a high school diploma or less. Additionally, insured participants had a lower median HALP score than uninsured participants. An array of confounding elements, including lifestyle factors and stress, could influence the HALP score among education backgrounds, and insurance statuses—prompting the need for further investigation as HALP gains utility.

It is also worth highlighting our participants' perceptions of their overall health. Participants who had rated their health as “poor” had a HALP score of 43.9, whereas those who reported “excellent” health had a score of 50.5. Although we cannot draw any definitive conclusions from these results, it emphasizes the need to continuously evaluate the relationship between patients' perceived health and their immunonutritional status.

### Cancer

Our study found that participants with a history of cancer did have a lower HALP score than participants who had never had a malignancy. A malignancy history lowers a participant's HALP score even when adjusting for sex, age, baseline CRP, anemia treatment in the last 3 months, kidney failure, and current cigarette use/smoking. To date, over 50 publications are using HALP to predict outcomes in cancer.

Most existing literature on HALP has discussed its predictive ability in cancer outcomes, including overall and recurrence-free survival. The only existing reviews on HALP include a 13,000-patient meta-analysis and one literature review. A low HALP score was found to predict cancer-specific, recurrence-free, progression-free, and overall survival (6). However, “low HALP” was defined as the number chosen by each author. This remained true across different tumor types and TNM staging. Farag et al. (2) summarized the findings of many of these individual papers and highlighted the heterogeneity of the “optimal threshold” at which HALP is most predictive for individual cancer types. Our study adds context to how these optimal thresholds may be interpreted and compared to the averages in the general US population with and without comorbidities across sex and other demographic factors.

Median HALP scores varied dramatically by cancer type. For example, participants with a history of ENT cancer had the lowest HALP scores (34.5), while participants with a history of lung cancer (49.1) had amongst the highest. This could be explained by the negative sequelae of head and neck surgery and radiation therapy, which may impair the swallowing mechanism. Consequently, patients may need to rely on a percutaneous endoscopic gastrostomy (PEG) tube, which could ultimately lead to significant nutritional consequences manifesting in a lower HALP score. Furthermore, participants with a history of leukemia and lymphoma exhibited notably low HALP scores (39.3), potentially due to their increased likelihood of undergoing targeted therapies to induce myelosuppression. The effects of chemotherapy and other treatment modalities on the HALP score must be better defined.

One limitation is that the NHANES only asks if a participant has had cancer instead of if a participant actively has a malignancy. This would skew the results in favor of healthier participants who survived their malignancy. However, a mere history of cancer significantly impacts a participant's HALP score implicating an element of chronicity in the HALP score. Also, the time interval between cancer diagnosis and participation in NHANES is unclear, as is the intensity of the prior treatment regimen (i.e., surgery, radiation, chemotherapy, immunotherapy, etc.). The lower HALP score for cancer patients is still significant, however, because many biomarkers that aim to provide insight into immunonutritional status are often acute-phase reactants. For example, CRP is considered a positive acute phase reactant, while albumin in the HALP score is regarded as a negative acute phase reactant. Elements of the HALP score do change acutely. However, it does seem that still, the HALP score has chronic prognostic ability in cancer, as highlighted by the plethora of existing literature on HALP and cancer outcomes. Our findings further underscore the need for a more comprehensive understanding of the factors influencing the HALP score in individuals with cancer.

### Kidney failure and dialysis

We further evaluated the prognostic value of the HALP score in predicting renal failure after accounting for clinicopathologic variables. In addition to revealing a significant negative relationship between HALP scores and kidney failure (β = −7.2, *p* < 0.001), our study demonstrates a modest predictive accuracy of the HALP score for kidney failure and dialysis that requires further research.

Chronic kidney disease (CKD) poses a substantial public health challenge, impacting over 843.6 million people globally in 2019 (7). As CKD progresses, the glomerular filtration barrier deteriorates, causing nutritional markers like serum albumin and BMI to decline and inflammatory markers such as CRP and IL-6 to increase due to Protein-Energy Wasting (PEW) (8, 9). This progression can ultimately lead to end-stage renal disease (ESRD), hemodialysis, and an increased risk of cardiovascular mortality. The neutrophil-lymphocyte ratio (NLR) and platelet-lymphocyte ratio (PLR) have been shown to predict inflammatory status and mortality in CKD patients (10–12). However, these markers do not account for the degree of protein wasting as CKD progresses. Low serum albumin levels have been included in nutritional predictive models, such as the malnutrition-inflammation score (MIS) and prognostic nutritional index (PNI), both associated with morbidity and mortality in CKD; however, the predictive value of these markers have yet to be fully validated (13–16). The HALP score's individual components are especially relevant to CKD patients due to the prevalence of anemia (i.e., low hemoglobin levels) secondary to decreased kidney erythropoietin (EPO) production (17). The NADIR-3 observational study revealed that patients with CKD who developed anemia had significantly poorer outcomes, including a more rapid progression to CKD stages 4–5 with higher rates of hospitalizations and even mortality (18). By incorporating nutritional and inflammatory markers, HALP may be able to alert of impending worsening CKD if used alongside other established models.

Moreover, our study showed that participants who underwent dialysis treatment in the past 12 months had a HALP score of 33.5 compared to 43.3 in those without dialysis treatment. This significant difference was further supported by identifying a HALP cutoff measure of 36.25 in predicting dialysis treatment. By leveraging this cutoff point in comparison to a generalized population, we hope that future researchers can develop and validate models to assess CKD status and dialysis initiation. Utilizing the HALP score in renal failure and dialysis patients may enable clinicians to identify patients who may benefit from more vigilant monitoring, nutritional support, and timely interventions, potentially improving treatment outcomes as HALP score thresholds are refined.

### Cardiovascular disease

Cardiovascular diseases (CVD) are the leading cause of death in the United States, accounting for 874,613 deaths in the US in 2019 (19). Our study evaluated the role of HALP in cardiovascular diseases, including CHF, high blood pressure, MI, CAD, high blood cholesterol, and strokes. We found a significantly lower median HALP score in self-reported CHF, stroke, and hypertension participants. Upon univariate analysis, participants with a history of CHF demonstrated a 5.6-point lower HALP score, and those with a history of stroke had a 3.7-point lower HALP score, which may inform future applications of HALP in CVD.

Currently, physicians use chronic inflammatory biomarkers, such as CRP, IL-6, and TNF-a, blood lipids, and insulin levels to predict the risk of developing CVD (20–22). As individual serum biomarkers, hemoglobin, albumin, lymphocyte, and platelet levels have been linked to CVD risk and prognosis. Both low and high hemoglobin concentrations correlate with increased CVD risk and mortality (23). Low serum albumin is associated with a higher risk of heart failure, hypertension, coronary artery disease, and stroke (24). Lymphocyte counts inversely relate to inflammation, indicating elevated cardiovascular risk and mortality (25, 26). Additionally, high LDL cholesterol, triglyceride levels, and low HDL cholesterol levels consistently contribute to increased CVD risk. Hyperlipidemia leads to shortened platelet survival and increased turnover, promoting platelet production (27), making elevated platelet counts valuable in predicting CVD risk and prognosis in patients. These components make using the HALP score potentially useful as supplemented by the insights NHANES has given us.

Although existing research into the HALP score in CVD in the clinical setting is limited, the HALP score has been used to predict patients' risk of ischemic stroke. A study showed that a lower HALP score indicates a higher risk of stroke, while a higher HALP score indicates a lower risk of stroke (28). Additionally, low HALP scores have also been found to be correlated with worse cognitive symptoms in people with acute ischemic stroke (29). Nevertheless, researchers have found that in patients with acute heart failure, the HALP score was inadequate in predicting an early or late prognosis for these patients (30).

Moreover, our analysis identified lower HALP scores among participants with elevated blood cholesterol and myocardial infarction, although these findings were not statistically significant. We also observed non-significantly higher median HALP scores in coronary artery disease participants. These insignificant results may be influenced by survivorship bias. Due to the cross-sectional nature of our study, participants who experienced previous cardiovascular events might have implemented lifestyle modifications and improved their overall health. Consequently, this could misrepresent the HALP values obtained during data collection, potentially masking the true relationship between the onset of these cardiovascular conditions and the HALP score.

### Asthma

Our study is the first to examine HALP's role in asthma, revealing that participants with asthma had lower median HALP scores, significant in univariate analysis. However, after adjusting for clinicopathological variables, the association between decreased HALP scores and asthma risk was insignificant. Asthma's development and pathophysiology are multifactorial, involving genetic, environmental, dietary, and other factors. Currently, established asthma prognostic markers include IgE, eosinophils, and fractional excretion of NO (FeNO) (31). Nutritional and inflammatory markers like the neutrophil-to-lymphocyte ratio (NLR) and dietary inflammatory index (DII), which consider nutrient and vitamin intake, may also be relevant in asthma risk prognosis (32, 33). However, the validity of these models in asthma remains unconfirmed. Moreover, recognizing the constraints of the NHANES dataset when evaluating our findings is crucial. Asthma, which commonly emerges in childhood or early adolescence, may have resolved in many participants by the time of data collection, a potentially substantial limitation. As the understanding of HALP scores progresses, their potential application as a prognostic instrument for asthmatic patients may become more apparent.

### Arthritis

Arthritis, affecting nearly a quarter of all Americans (34), has inflammatory or non-inflammatory etiologies. Our study investigated HALP's role in both classifications, finding lower median HALP scores in participants with rheumatoid arthritis. This aligns with prior research indicating that the controlled nutritional status score (CONUT) and the nutritional risk index (NRI), which include nutritional markers such as albumin, body weight, and lymphocyte count, are associated with increased all-cause mortality in rheumatoid arthritis (35).

We also observed a decreased median HALP in participants with osteoarthritis. Although osteoarthritis was once considered a “wear and tear” disease, recent research highlights the role of inflammatory processes, including activated innate immune cells, elastases, and IL-6 (36–39). Early studies suggested an association between C-reactive protein levels and worse osteoarthritis progression; however, recent literature has shown that CRP and the platelet-to-lymphocyte ratio (PLR) are not strongly associated with osteoarthritis (40). Instead, neutrophil-to-lymphocyte ratio (NLR) and neutrophil-to-monocyte ratio (NMR) are elevated in osteoarthritis patients (41). Despite our results finding significance in univariate, but not multivariate, HALP may still offer prognostic value as it is further investigated alongside other inflammatory biomarkers such as NLR and NMR in the future.

### Thyroid

Our findings reveal that participants with self-reported thyroid issues exhibit significantly lower median HALP scores on univariate analysis; however, this association lost significance in the multivariate analysis. The role of HALP as a predictive tool for thyroiditis has not yet been explored. Previous research has found serum acute phase reactant C reactive protein (CRP) elevated in these patients (42). Additionally, the platelet-to-lymphocyte ratio (PLR) has also been found to be increased in cases of thyroiditis (43). Adding nutritional markers could provide improved efficacy compared to PLR, as patients with thyroiditis have also been shown to have decreased hemoglobin (44). However, after accounting for all other clinicopathological variables, our results found no significant association of HALP with the risk of thyroid problems. A considerable limitation here is the lack of clarity regarding hyper- vs. hypothyroidism. It has been reported that hypothyroidism was present in 4.6% of the population (with a higher presence of subclinical than clinical hypothyroidism), while hyperthyroidism was present in 1.3% of the population (with the almost equal presence of subclinical and clinical hyperthyroidism) (45).

### COPD/emphysema

Participants with COPD/emphysema exhibited lower median HALP scores, which were significant in univariate analysis but not multivariate analysis. Previous research has found reduced hemoglobin and albumin levels in COPD patients (46, 47), increasing interest in the HALP score for predicting acute exacerbation risks in this population. Lower HALP scores have been linked to higher ICU mortality rates during acute exacerbations, suggesting the HALP score may be a prognostic indicator in COPD patients (48). Our study corroborates these findings, as participants with COPD demonstrated lower HALP scores, potentially highlighting HALP's role as an inflammatory marker in COPD. Further research is necessary to elucidate the relationship between HALP and COPD. Yet, our study and prior investigations indicate the potential utility of the HALP score as a prognostic tool for COPD management and treatment.

### Cigarette smoking

Interestingly, our results show that cigarette smokers had a higher HALP score than non-cigarette smokers, 55.0 (40.9–71.5) and 49.1 (36.9–65.4) (*p* < 0.001), respectively. Individual differences, including genetics and other lifestyle factors, could explain this difference among participants. However, multiple studies have shown markedly increased hemoglobin levels (49). Within our study population, cigarette smokers were found to have a median hemoglobin (g/dL) of 14.5 (IQR: 13.4–15.4), while non-cigarette smokers had a median hemoglobin of 14.2 (IQR: 13.1–15.1), this difference was statistically significant (*p* < 0.001). This could be attributed to the presence of carbon monoxide in cigarette smoke. When inhaled, carbon monoxide forms carboxyhemoglobin within red blood cells, a compound that cannot carry oxygen. As a result, the body experiences peripheral hypoxia, which can trigger a compensatory response to maintain adequate oxygen transportation. This response involves an increase in hematocrit and hemoglobin levels, ensuring sufficient oxygen reaches tissues despite the diminished oxygen-carrying capacity caused by cigarette smoking (49). Given the global prevalence of cigarette smoking, accounting for a patient's smoking history is crucial when assessing the HALP score and other immunonutritional biomarkers.

## Advantages and limitations

There are several advantages to our study. This study is the first to provide insights into the HALP score from a sample representative of the diverse ethnic and demographic makeup of the adult population in the United States. Our findings bolster the expanding body of knowledge on HALP, paving the way for future research. It is crucial, however, to acknowledge the inherent limitations of our study.

The cross-sectional nature of our analysis precludes any inference of causality. Moreover, our results may be influenced by several confounding variables, such as compliance with recommended treatment for comorbidities and medication use. These variables may impact participant HALP scores and should be considered when interpreting our findings. Furthermore, limited information was available concerning the chronicity of each participant's comorbidity. Therefore, we could not account for the association between the disease duration and the HALP score.

Participants self-reported their comorbidities and health information, which could introduce recall bias and exclude treatment influences. For instance, a history of well-controlled hypertension may have very different implications than severe uncontrolled hypertension. Regarding cancer, the NHANES specifically ask if the participant has ever had cancer, as opposed to differentiating active cancers. However, it was notable that self-reported general health status correlated well with the calculated HALP score, legitimizing the patient-reported methodology of NHANES. Additionally, laboratory values were only collected at one-time point, preventing us from examining HALP score changes over time and in response to treatment for various comorbidities. Our ROC analysis revealed that the HALP score had limited predictive capability, suggesting that HALP may function as an independent comorbidity and should be reported independently when evaluating a patient's health status as a predictor of outcomes. Finally, the data collected from 2019–2020 was unavailable due to the coronavirus (COVID-19) pandemic, which included data supplemented by a weighted method.

## Conclusion

Examining the baseline HALP score in the U.S. adult population yielded results that displayed the HALP score through a new lens; a shift from disease-specific interpretations to a population-based understanding. Our findings unveil significant associations between HALP scores and a diverse array of demographic, socioeconomic, and health conditions, offering valuable insights into the potential implications of this score in clinical settings. As HALP correlated significantly with various comorbidities, it is reasonable to propose that HALP should be envisioned and included as an entirely separate “comorbidity” or even “performance index” with perhaps farther-reaching prognostic capabilities than any other single comorbidity alone. This is similar to other established performance indices such as the American Society of Anesthesiology (ASA) score, Charlson Comorbidity Index (CCI), and Karnofsky Performance Scale (KPS). The median HALP score among this study's representative population was 49.0, with varying median scores across different groups and normal reference ranges for males and females. Laying the foundation for future research, this study looks to ultimately supplement the determination of clinical thresholds of HALP and help clinicians assess their patients' immunonutritional status and care.

## Data availability statement

The original contributions presented in the study are included in the article/Supplementary material, further inquiries can be directed to the corresponding author.

## Author contributions

RA and MW conceived the idea for conducting this study with support from CF. CF and RA contributed to statistical analysis, while VX and AD conducted a literature review and contributed to the writing with support from OG and MW. All authors contributed to editing equally.
