# Supplementary figures and images for: Evaluating the baseline hemoglobin, albumin, lymphocyte, and platelet (HALP) score in the United States adult population and comorbidities: an analysis of the NHANES
Source: Front Nutr. 2023 May 18;10:1206958. doi: 10.3389/fnut.2023.1206958 (PMC10240525; doi:10.3389/fnut.2023.1206958)

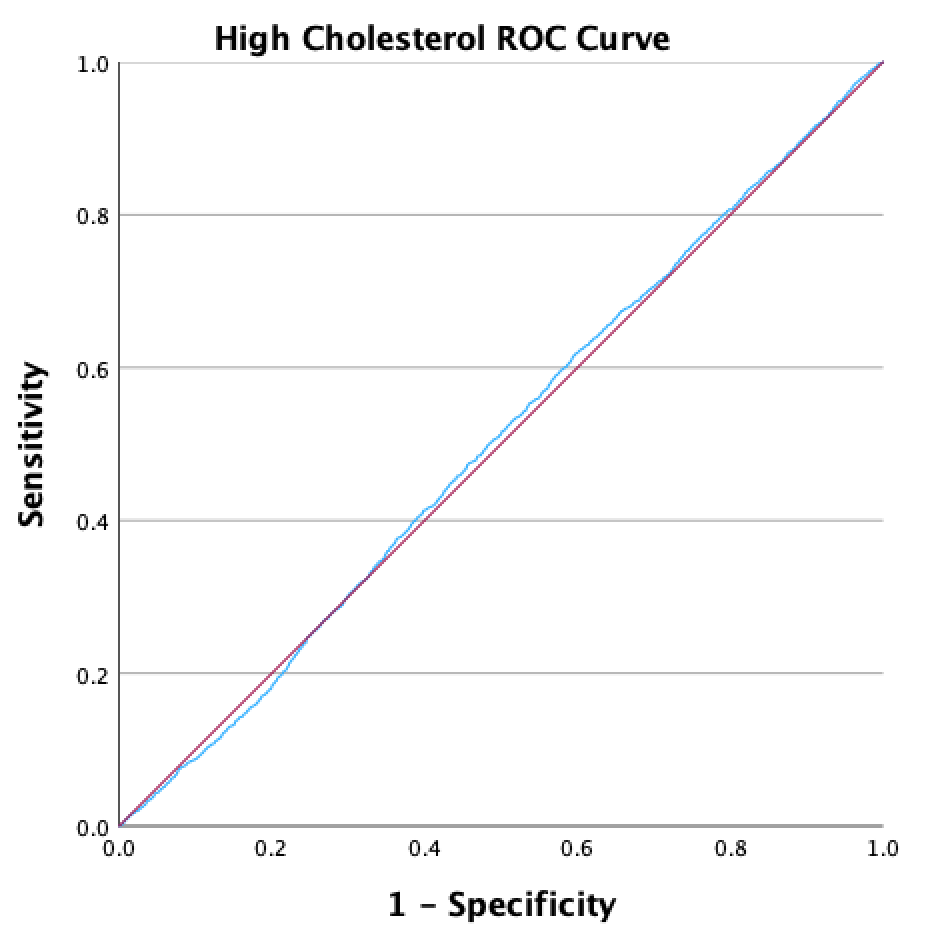


**
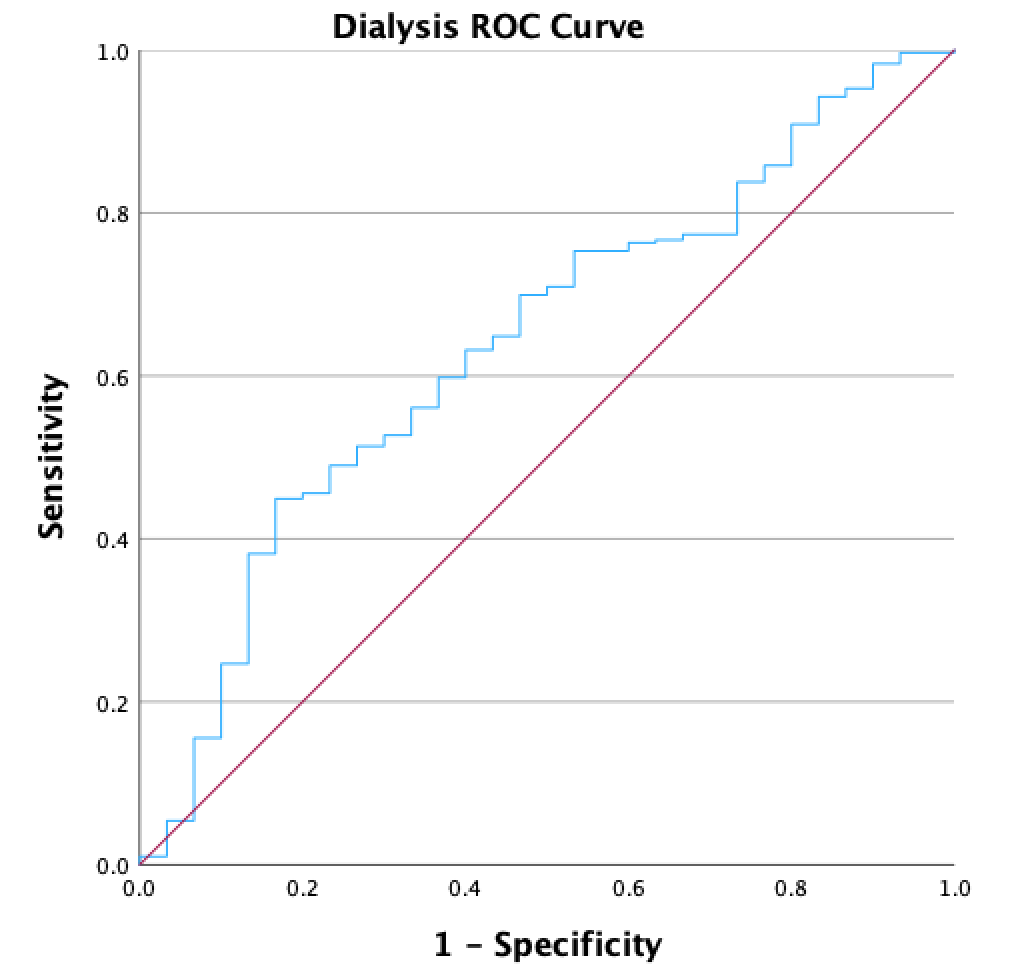
**
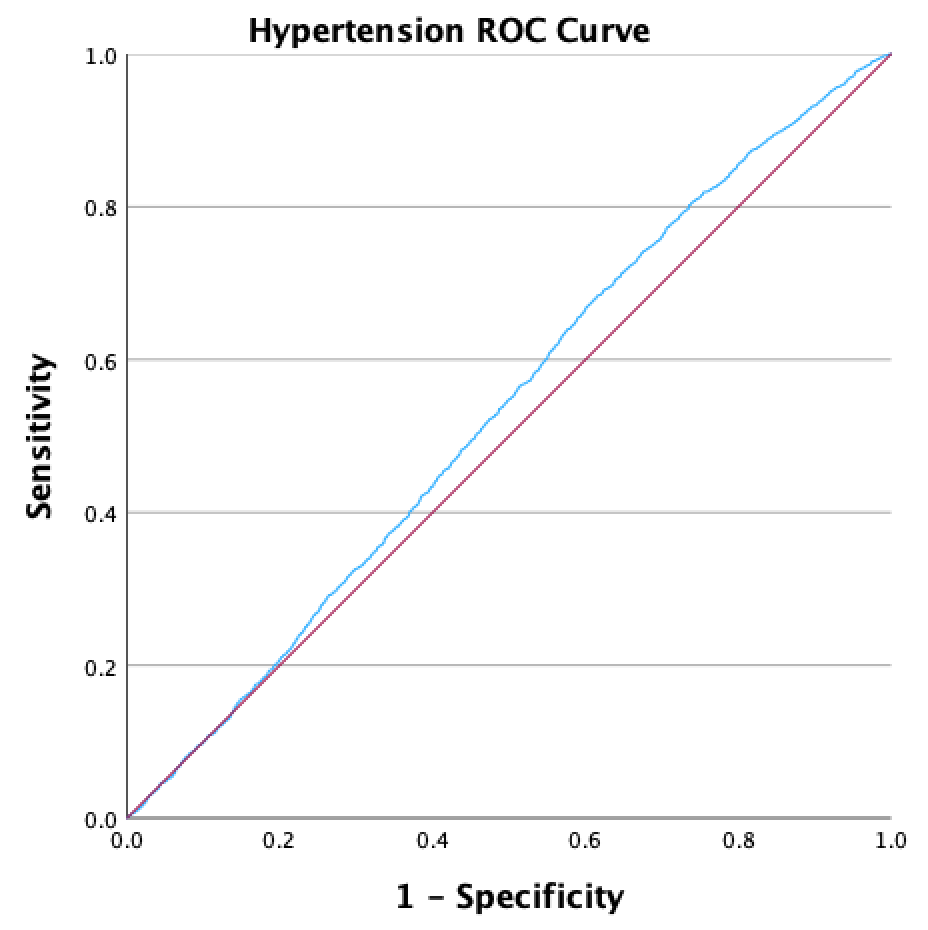

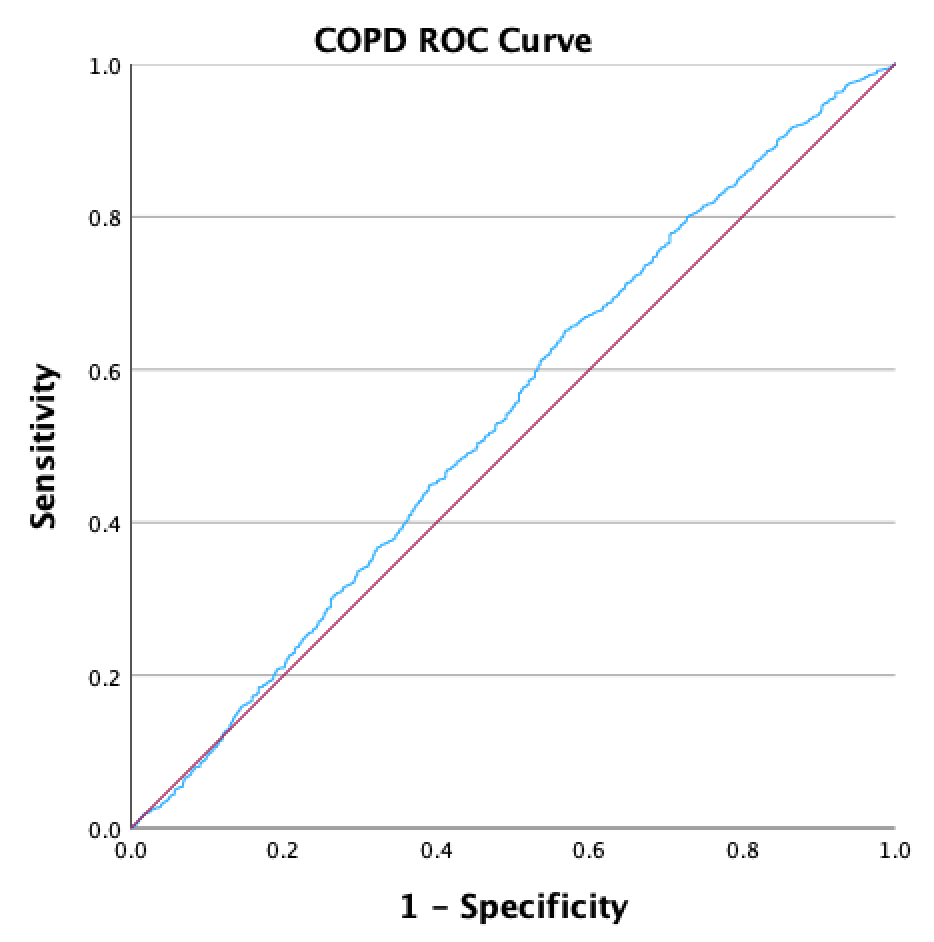

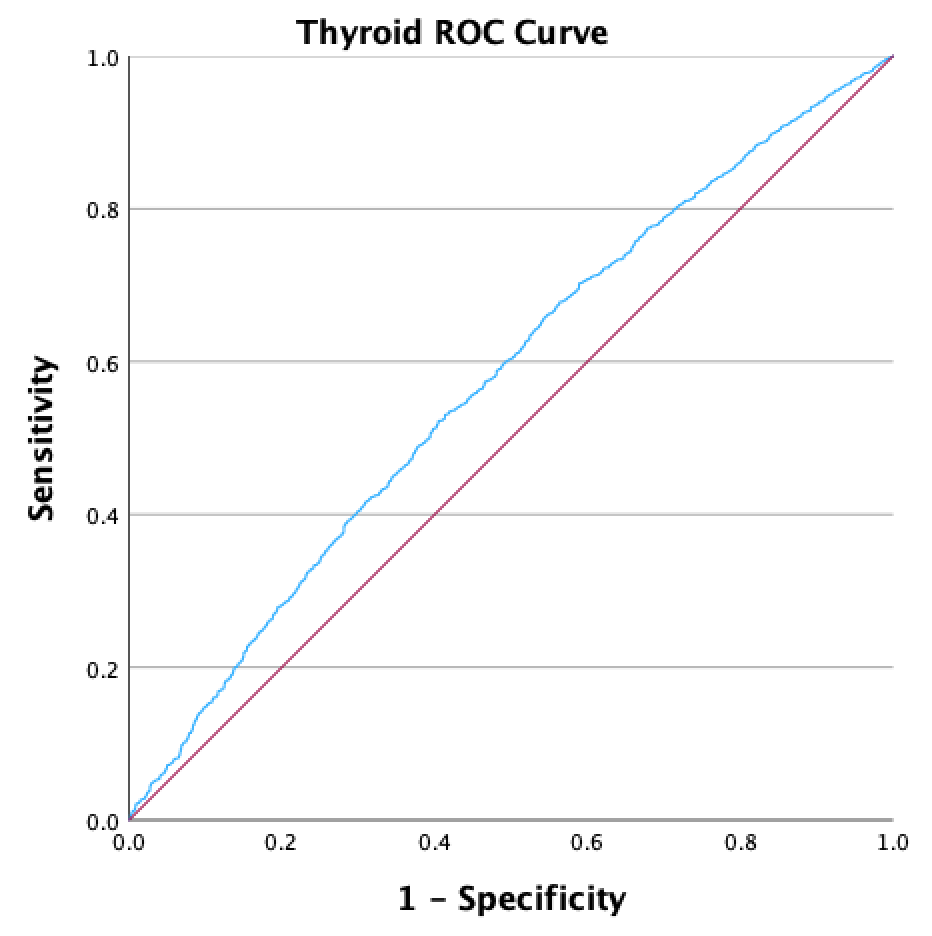

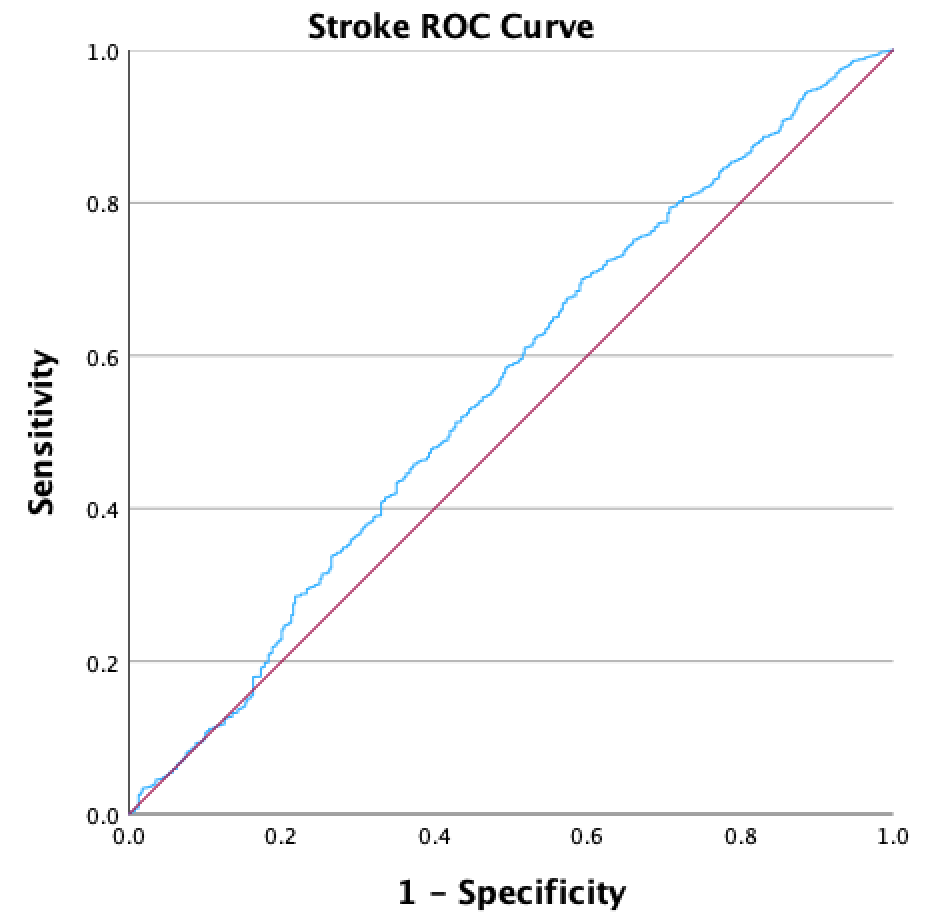

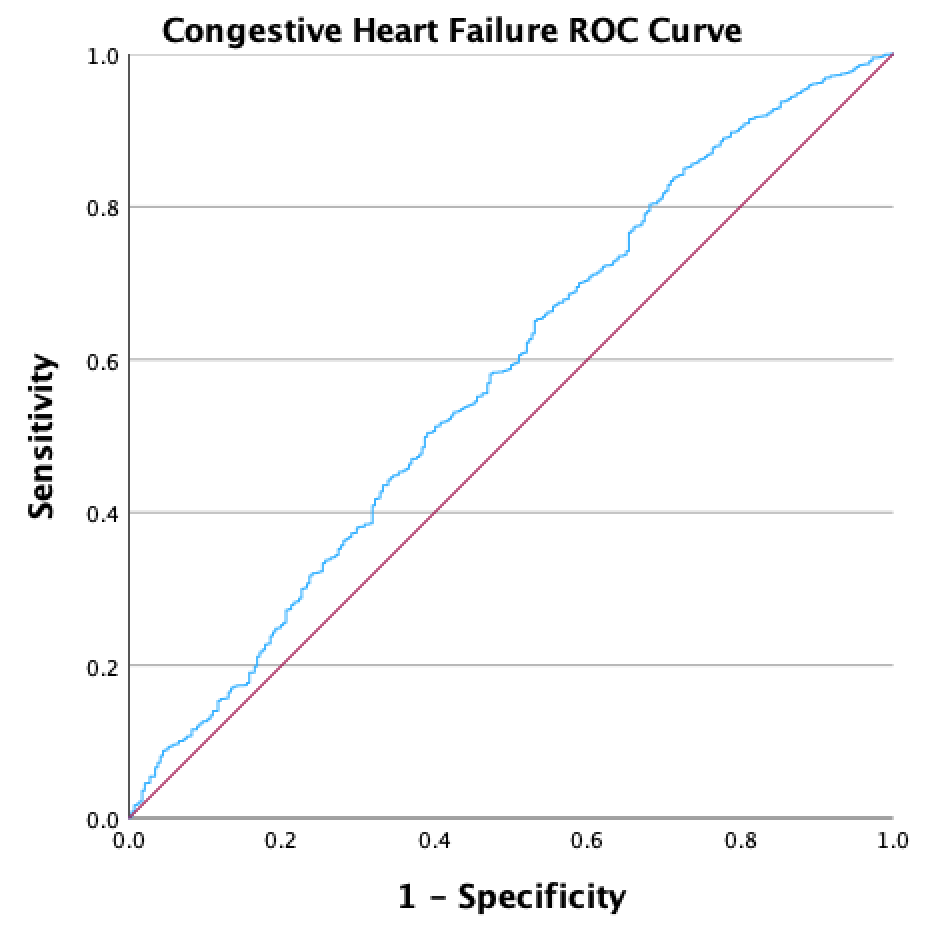

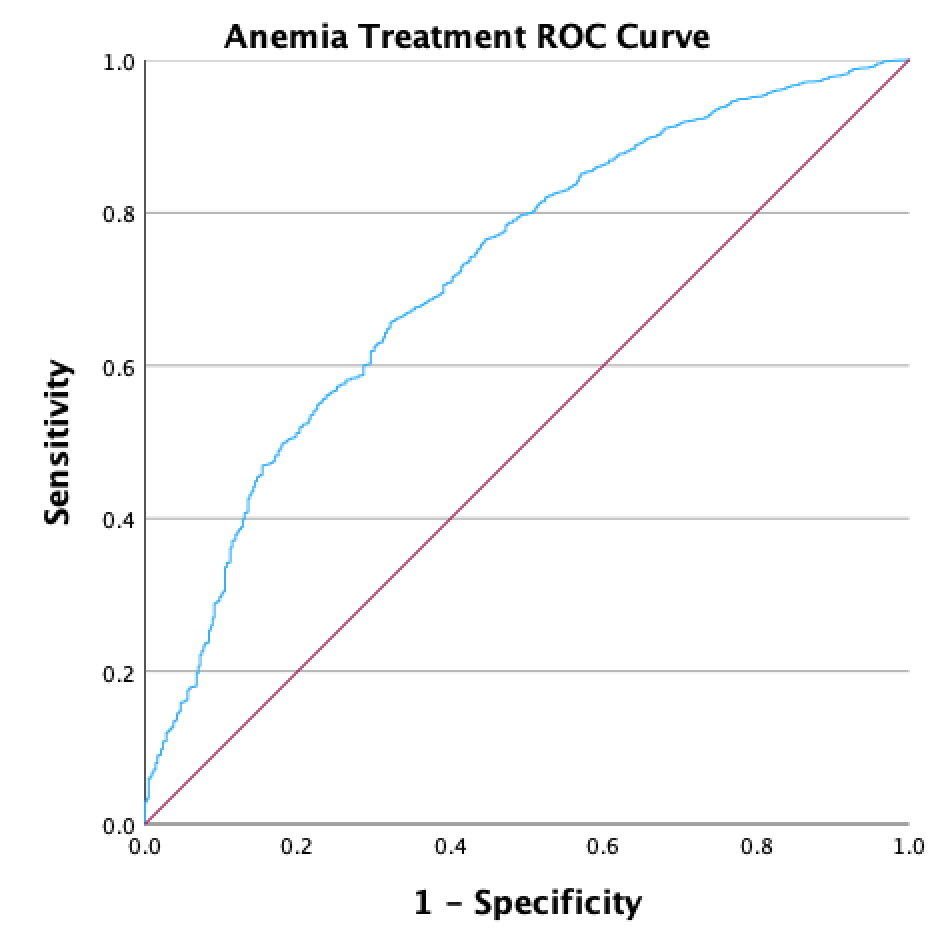

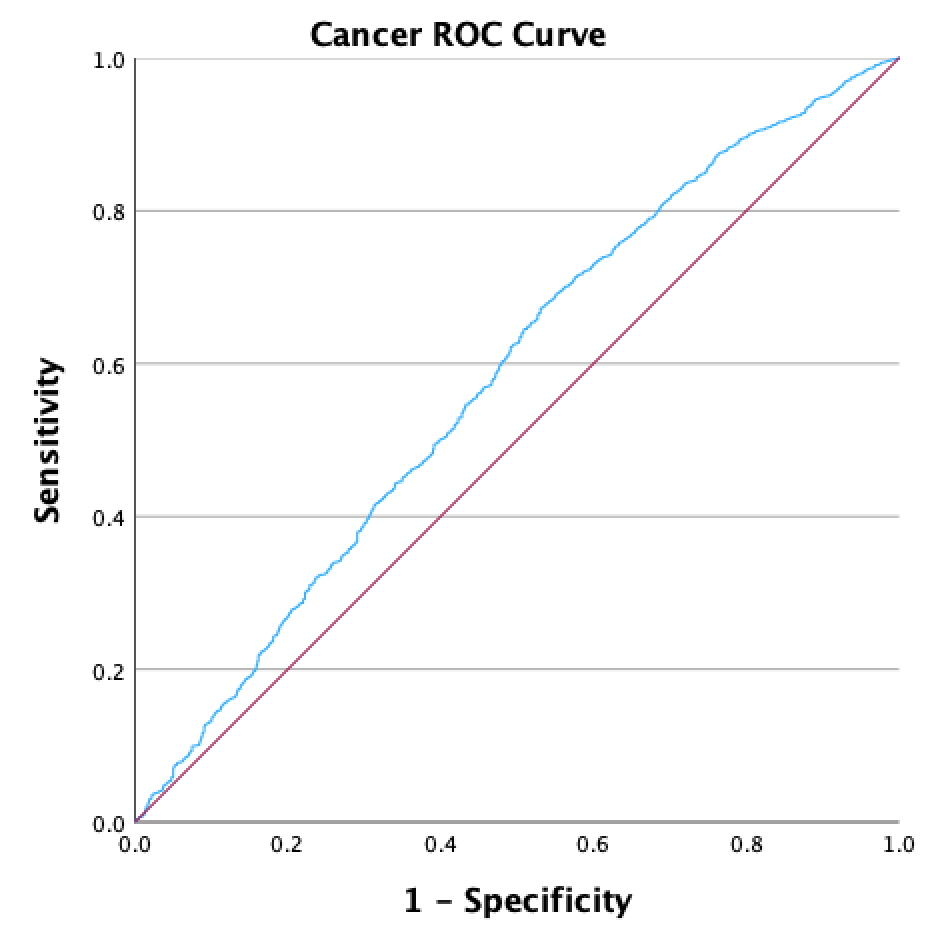

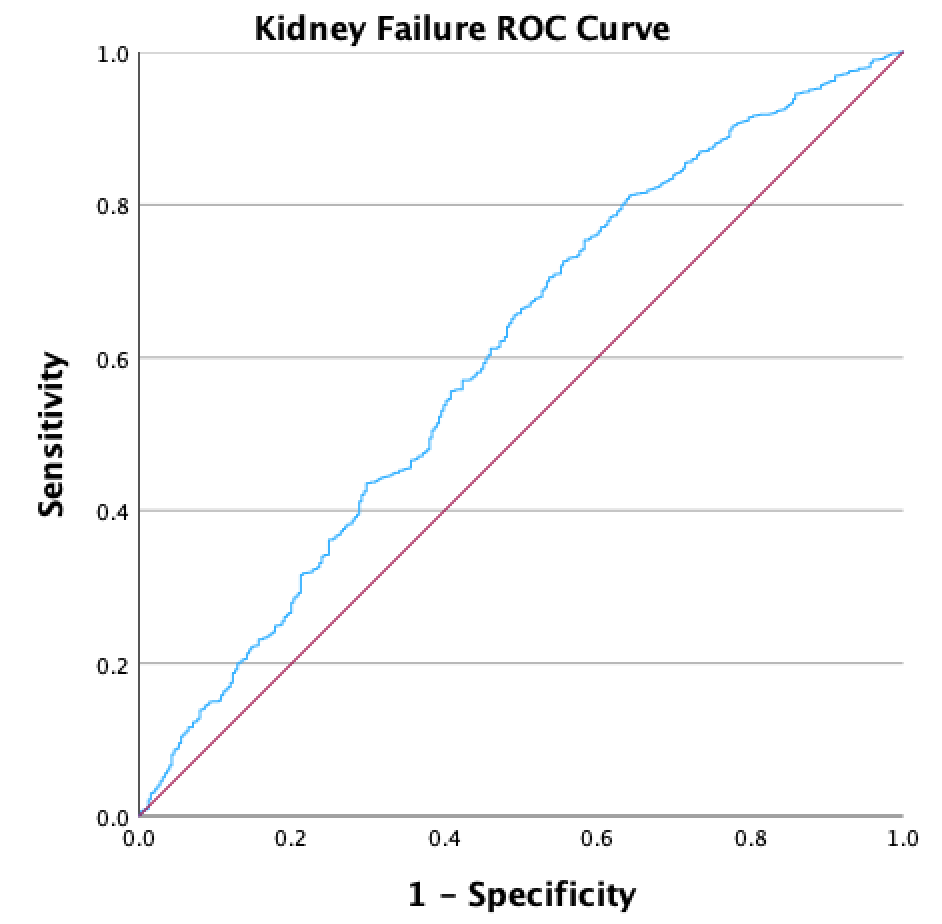

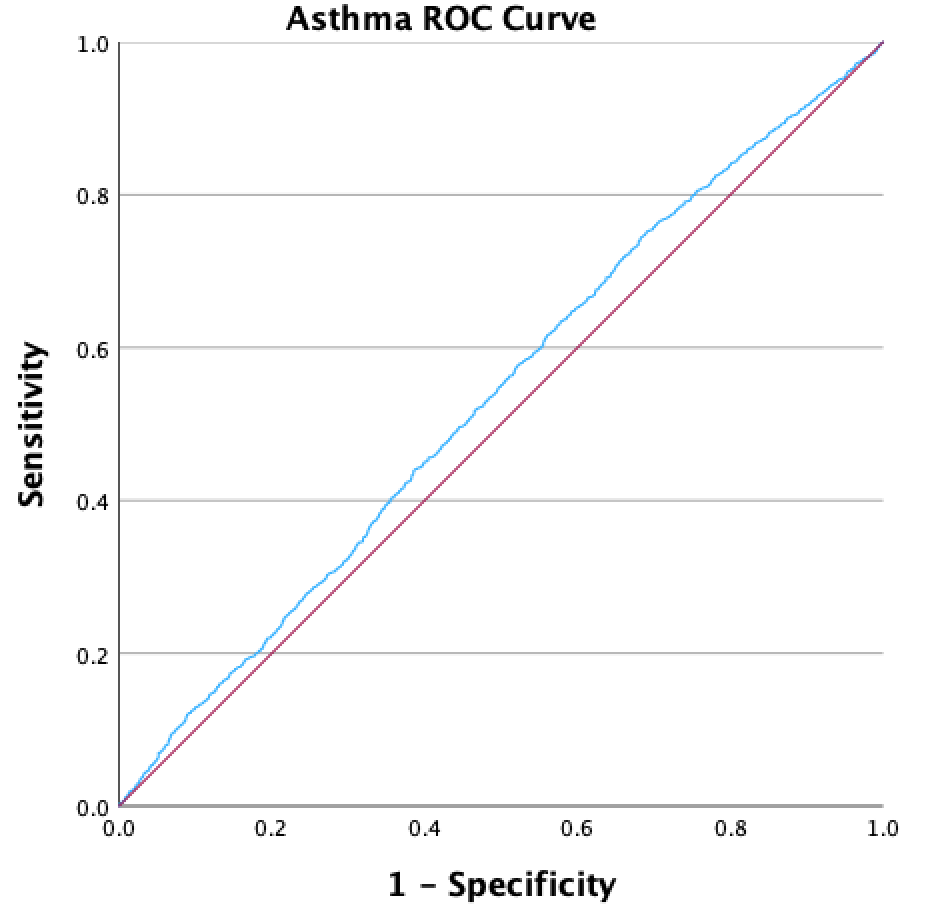

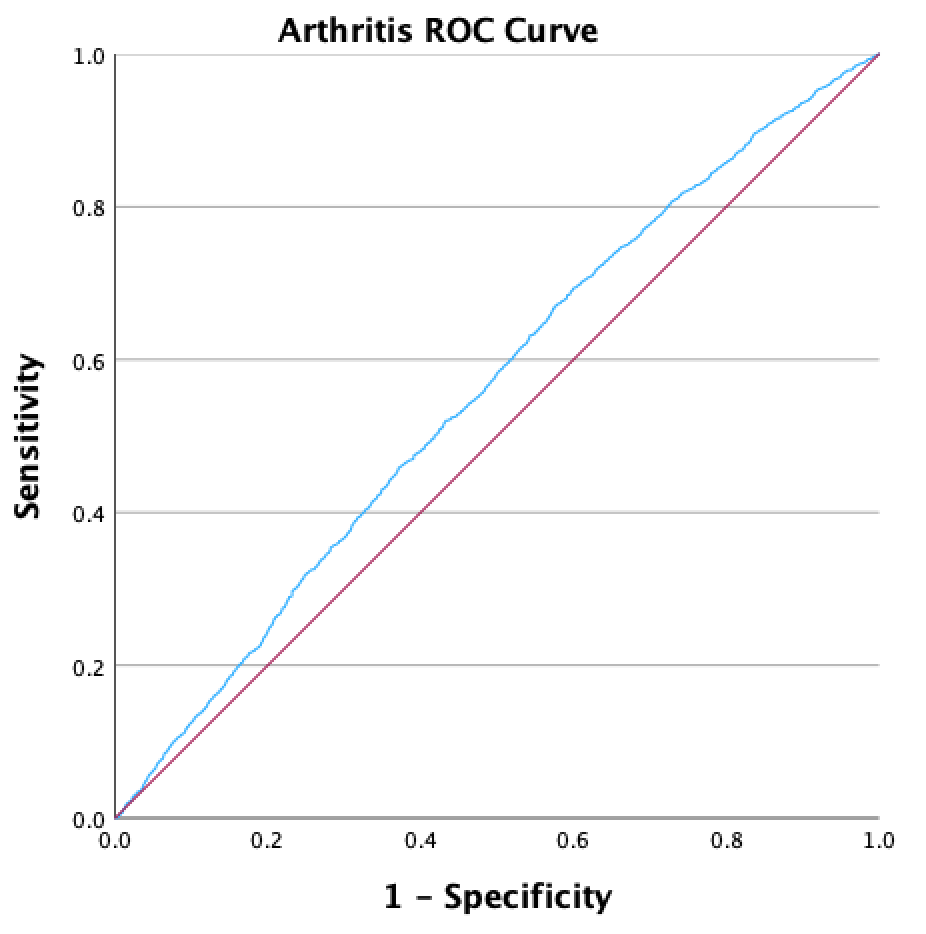

Supplement: Supplementary file 1 [file Data_Sheet_1.docx]
